# Association between overweight, obesity and sleep duration and related lifestyle behaviors is gender and educational stages dependent among children and adolescents aged 6-17 years: a cross-sectional study in Henan

**DOI:** 10.1186/s12889-022-14068-x

**Published:** 2022-08-31

**Authors:** Yiran Wang, Shuying Luo, Yuwei Hou, Kaijuan Wang, Yaodong Zhang

**Affiliations:** 1 Henan Key Laboratory of Children’s Genetics and Metabolic Diseases, Children’s Hospital Affiliated to Zhengzhou University, Henan Children’s Hospital, Zhengzhou Children’s Hospital, Zhengzhou, Henan Province China; 2 Department of Epidemiology and Health Statistics, College of Public Health, Zhengzhou University, Zhengzhou, Henan Province China

**Keywords:** Overweight/Obesity, Sleep duration, Gender, Educational stages, Children/adolescents

## Abstract

**Background:**

To investigate the associations between overweight, obesity and sleep duration and related lifestyle behaviors in children and adolescents at different gender and educational stages.

**Methods:**

A cross-sectional study comprising 18723 children and adolescents with a stratified cluster sampling method of Henan Province was conducted in 2019. A self-reported questionnaire was used to collect the information about demographic characteristics as well as sleep and lifestyle behaviors. Anthropometric measurements (height and weight) were taken and body mass index was computered as an indicator of overweight and obesity. The Chi-square test, one-way analysis of variance and multiple logistic regression were used to data analysis.

**Results:**

Among the respondents, 12657(67.6%) were with normal weight, 3711(19.8%) were overweight and 2355(12.6%) were obesity. The average age of the participants was 12.6 years old. The proportion of overweight and obesity in the 10191 boys was 18.7% and 14.2% respectively. The proportion of overweight and obesity in the 8532 girls was 21.2% and 10.6% respectively. In trend analyses, sleep duration at different gender found with the decreased of the sleep duration, the proportions of overweight/obesity in boys and girls were gradually increased (P_trend_<0.05). In the adjusted logistic regression models, the results showed stratified by gender, compared with the recommended sleep duration group, students with very short sleep duration and short sleep duration showed an increased OR_adj_ of 2.56 and 2.13 in boys, 2.34 and 2.09 in girls respectively. According to different educational stages, those in very short sleep duration and short sleep duration showed an increased OR_adj_ of 2.15 and 1.69 in primary school, 2.26 and 1.58 in middle school, 2.23 and 1.51 in high school respectively.

**Conclusions:**

Children and adolescents with very short sleep duration and short sleep duration may increase the risk of overweight/obesity, the association differed based on the gender-specific and educational stages-specific. Gender and educational stages should be regarded as specific characteristics for the effects on overweight/obesity in Henan Province.

## Background

Overweight and obesity in children and adolescents have become a serious public health problem. It’s worrying that the prevalence of overweight and obesity among children and adolescents has increased all over the world, and very young children were affected-about forty-one million children aged under five were overweight or obese in 2016 globally, with almost half of them living in Asia [1–5]. Despite public health efforts, the proportion of overweight/obesity has continued to rise dramatically in both developed and developing countries during past few decades, and China is no exception [6–8]. According to data from the Report on Childhood Obesity in China in 2017, the number of children and youth with overweight or obesity increased from 6.15 million in 1985 to 34.96 million in 2014 [9, 10]. Several studies [11–13] showed that overweight/obesity has serious short-and long-term consequences for physical and psychosocial health, including cardiovascular diseases, diabetes, hypertension, stroke, metabolic syndrome, etc. Additionally, overweight/obesity can also affect children’s and adolescents’ mental health and psychosocial development, leading to poor socialization, low self-esteem and negative self-evaluation and reduce educational attainment. In addition to the influences on the health of individuals, overweight/obesity also puts a major economic burden on governmental health organizations.

With rapid economic development, people’s standards of living have vastly improved and their lifestyles have undergone tremendous changes over the past few decades in China. Children and adolescents rising levels of overweight/obesity have also been attributed to the country’s recent socioeconomic growth. Adolescence is an important stage of life for the development and maintenance of health and risk behaviours, many of which are associated with overweight and obesity. Therefore, understanding factors associated with children and adolescents overweight/obesity is critical for addressing this pressing public health concern. The correlates of overweight/obesity in children and adolescents are numerous and complex, but several known factors that could contribute to an increase in the proportion of overweight/obesity, including a lack of physical activity, sedentary behaviors, increased screen time, food availability and parental influences [14–16]. Conventionally, studies on the influence factors of overweight/obesity in children and adolescents mostly focus on the eating habits and physical activity. In recent years, some studies have shown that sleep duration has been proposed as a possible risk factor of overweight/obesity, but these studies may somewhat inconsistent considering the changes in study design, characteristics of the population or classification criteria.

Sleep is one of the modifiable behaviors and plays an important role in growth and development in children and adolescents overweight/obesity. Short sleep duration is very common in modern society, with approximately one-third to two-thirds of children and adolescents complain about short sleep. Many studies have explored the relationship between sleep duration and overweight/obesity [17–20]. Several cross-sectional studies have reported that short sleep duration is associated with childhood overweight/obesity. With regard to prospective studies, a meta-analysis that reviewed 22 longitudinal studies revealed twice the risk of obesity in subjects reporting short sleep. Although the relationship between sleep duration and overweight/obesity has been extensively investigated in children and adolescents, including in Chinese children, but the requirements for daily sleep duration may also vary due to the different gender, so gender differences in sleep duration in an important factor that should be taken into account. Additionally, educational stages-specific factors that can influence the proportion of overweight/obesity remains obscure in the Chinese children and adolescents population. Moreover, overweight/obesity is not only affected by sleep duration, but is also modulated by physical activity, diet habits and other behavioural factors that are difficult to quantify. Inequalities in the prevalence of overweight and obesity were documented in China-a greater prevalence increase was observed among higher socio economic status (SES) children. For example, in Beijing, the capital city of China, the prevalence of overweight and obesity among preschool children was 19.44% in 2016 in Shijingshan District [21]. By reviewing the published literatures, there were few studies on the association between sleep duration and overweight/obesity in the children and adolescents based on the gender and educational stages simultaneously in Henan Province. In addition, Henan as the major province in the central plains in China, the children and adolescents always with their special living and eating habits in their growth environment. In order to make the study population more representative and provide the evidence for preventing overweight and obesity among children and adolescents at different gender and educational stages in Henan Province. Therefore, it is necessary to carry out the regional research in Henan Province. Given this background, we explored whether the relationship between sleep duration and related lifestyle behaviors and overweight/obesity is gender- and educational stages-dependent in the Chinese population. We conducted a cross-sectional study among children and adolescents aged 6-17 years in the urban area of Henan Province. Our study aimed to investigate (i) the up-to-date proportion of overweight/obesity, (ii) stratified analysis the proportion of potential overweight/obesity influencing factors among children and adolescents according to gender-specific and educational stages-specific and (iii) the associations between overweight/obesity and sleep duration and related lifestyle behaviors regarding gender-specific and educational stages-specific differences.

## Methods

### Study population

This survey was a cross-sectional study. A three-stage random cluster sampling method was used to find participants in this survey. In the first stage of sampling, eight cities were selected from the eastern, western, central, southern and northern regions of Henan Province using the method of probability proportional to size. In the second stage of sampling, six schools were randomly selected from all schools in each selected city using the same method. In the third stage of sampling, six classes were randomly selected in each selected school using the same method. All of the students in the selected classes were invited to participate in the survey. The number of classes to be recruited was determined by a sample size extimation formula: $N=K\times \frac{{\mu_{\alpha}}^2p\left(1-p\right)}{\delta^2}$_._ Assuming the prevalence of overweight/obesity among children and adolescents was 22.8% (estimated through a pre-experimental study), a two-sided significance level of 5% (u_α_=1.96), a minimally detectable rate difference(δ) of 1.5%, and an expected 10% non-response rate and a 2.0 design effect of cluster sampling, the number of children and adolescents needed was calculated. A total sample of 18723 students (10191 boys and 8532 girls) aged 6-17 years were recruited in this study. They were divide into three groups, the normal weight group (*n*=12657), the group with overweight (*n*=3711) and the group with obesity (*n*=2355). All the research students were Henan Province resident population who could provide detailed home address, excluding morbidly obese children and adolescents and those with lack of sleep duration information.

### Ascertainment of variables

A specially designed and standardized questionnaire was designed to collect information on socio-demographic characteristics, daily sleep duration, daily physical activity, daily sedentary behaviors and daily eating habits associated with children and adolescents overweight/obesity. Questionnaires were distributed in class, and students were instructed to give the form to their parents. Any question or confusion was clarified to ensure that every student understood all of the items, and parents were provided with instructions on answering the questionnaire to prevent inaccurate information. Trained investigators would collect the questionnaires from each class and quality control would be performed. To ensure the reliability of the information, we routinely examined the questionnaires on the survey day and checked for missing items and logical errors. When data was found to be inconsistent, we would list the numbers of the questionnaires, record names, variable names and error categories to facilitate future checks and corrections.

In this study, we mainly evaluated six lifestyle-related parameters, including sleep duration, physical activity, internet surfing, watching TV, playing on phone and eating habits before sleeping. Daily reported total sleep duration was assessed through self-reported recordings as follows: on average, how many hours and minutes did you sleep on a typical day during the past seven days? In this study, the sleep duration was divided into four levels according to the National Sleep Foundation (NSF) guideline for age-specific sleep recommendations: very short, short, recommended and long sleep duration [22]. For children aged 6 to 13 years, sleep duration < 7h/d was classified as very short, within 7 to 8 h/d as short, within 9 to 11 h/d as recommended, and more than 11 h/d as long. For children aged 14 to 17 years, sleep duration < 6h/d was classified as very short, within 6 to 7 h/d as short, within 8 to 10 h/d as recommended, and more than 10h/d as long. The recommended sleep-duration group was considered the reference group in this study. Daily time spent in sedentary behaviors including internet surfing, watching TV or playing on phone was investigated with children and adolescents to report the amount of sedentary behaviors time (in hours) they spent during the past seven days, about a half hour, 1h, 2h,or 3h or more. Then we would calculated the time by multiplying the weekly frequency of participation with the duration per bout of participation in sedentary activities, and then dividing by seven. Then, we used the threshold of 2h/day proposed by current scientific evidence and guidelines, students were classified as exceeding (>2h/d) or not exceeding(≤2h/d) the recommended daily time spent in sedentary behaviors. Physical activity was investigated by the question: how many hours each day do you usually spend in physical activity? Students recorded time spent in hard (i.e., jogging, team sports) and moderate (i.e., walking, biking to school) physical activity on each of the previous seven days. Responses were also dichotomized into two groups (>2h/d vs.≤2h/d). Eating habits before sleep was assessed by asking students if they had eating habits before sleep daily, the types of foods eaten including consumption of red meat, puffed food, fresh fruits, fresh vegetables or milk. The responses were dichotomized into two groups (yes vs. no).

### Anthropometric measurement

Children and adolescents height and weight were measured by the well-trained health professionals. Height was measured to the nearest 0.1 cm with subjects wearing no shoes, and weight was measured to the nearest 0.1 kg with subjects wearing lightweight clothing. Two measurements (measurement error≤1mm) were obtained and the average was used for the analysis. Body mass index (BMI) was calculated as weight (kg) divided by height squared (m^2^). Overweight and obesity were defined using age- and gender-specific BMI cutoff points issued by the National Health Commission of the People’s Republic of China.

### Statistical analysis

Children and adolescents baseline descriptive characteristics with continuous variables and categorical variable were all presented as mean±standard deviation value and frequencies and percentages respectively. The Chi-square test for categorical variables and one-way analysis of variance (ANOVA) were conducted to evaluate differences among different groups. As a remarkably different proportion and incidence of overweight/obesity between boys and girls was observed, stratified analysis based on gender was performed to examine the proportion of potential overweight/obesity influencing factors among children and adolescents according to gender-specific. Stratified analysis based on educational stages-specific was also conducted to evaluate whether there was effect modification of overweight/obesity. To further explore gender and educational stages differences in the relationship between sleep duration and overweight/obesity in different groups, we also conducted trend test analysis stratified by the gender-specific and educational stages-specific respectively.

Considering that there still were nonmatching variables among different groups apart from age, gender, and residence region, we carried out unconditional multiple logistic regression models to estimate the associations between overweight/obesity and sleep duration and related lifestyle behaviors regarding gender and educational stages differences. Collinearity between potential confounding variables was examined using Spearman rank-order correlation for continuous variables or Chi-square test for category variables. We generated odds ratios (ORs) and 95% confidence intervals (CIs) after adjusting for age, residence region, height, weight, parental age, parental BMI and parental educational level according to the gender-specific and educational stages-specific groups. These covariates were chosen on the basis of the preliminary statistical results of the present study. All statistical analyses were conducted with IBM software SPSS (version 21, Chicago, IL, USA). All statistical tests were two-tailed and considered to be statistically significant at *P* value less than 0.05.

## Results

### Baseline characteristics of the study population

The baseline characteristics of the study population were presented in Table 1. A total of 18723 children and adolescents were included in the final analysis. Among the respondents, 12657(67.6%) were with normal weight, 3711(19.8%) were overweight and 2355(12.6%) were obesity. The average age of the participants was 12.6 years old. The proportion of overweight and obesity in the 10191 boys was 18.7% and 14.2% respectively. The proportion of overweight and obesity in the 8532 girls was 21.2% and 10.6% respectively. In comparison with students in middle school and high school, the proportion of overweight and obesity among students in primary school was highest. There were no difference between the three groups with respect to the internet surfing (*P*=0.612) and watching TV (*P*=0.079). Meanwhile, all other considered characteristics tested with statistically significant difference among the three groups (all *P*<0.05).

**Table 1:** Baseline characteristics of the study population [n(%)]

Variables	Total (*n*=18723)	Normal weight (*n*=12657)	Overweight (*n*=3711)	Obesity (*n*=2355)	*P*-value
Age (years)	12.6±3.1	12.5±4.2	12.9±3.2	13.1±3.5	<0.001
Gender					<0.001
Boys	10191	6842 (67.1)	1902 (18.7)	1447 (14.2)	
Girls	8532	5815 (68.2)	1809 (21.2)	908 (10.6)	
Residence region					<0.001
Urban	13347	8484 (63.6)	3122 (23.4)	1741 (13.0)	
Rural	5376	4173 (77.6)	589 (11.0)	614 (11.4)	
Educational stages					<0.001
Primary school	7122	3488 (49.0)	2492 (35.0)	1142 (16.0)	
Middle school	5823	4590 (78.8)	619 (10.6)	614 (10.6)	
High school	5778	4579 (79.2)	600 (10.4)	599 (10.4)	
Height (cm)	166.3±4.4	166.2±4.4	166.1±4.2	167.2±4.6	<0.001
Weight (Kg)	59.4±4.1	49.3±3.8	57.5±4.3	69.4±4.7	0.003
BMI (kg/m^2^)	25.4±2.9	21.3±2.7	25.5±3.7	34.9±3.5	<0.001
Paternal age	41.2±2.0	41.1±2.0	41.2±3.7	41.0±2.0	0.001
Paternal BMI (kg/m^2^)	26.5±2.7	20.9±1.8	25.1±3.2	32.5±1.7	<0.001
Paternal education level					<0.001
Middle school or below	5220	3725 (71.4)	812 (15.5)	683 (13.1)	
High school	5159	3536 (68.6)	986 (19.1)	637 (12.3)	
University or above	8344	5396 (64.7)	1913 (22.9)	1035 (12.4)	
Maternal age	40.0±3.0	40.1±3.0	39.7±3.0	40.3±3.1	<0.001
Maternal BMI (kg/m^2^)	25.8±1.9	21.1±1.1	25.2±2.9	31.6±2.3	<0.001
Maternal education level					<0.001
Middle school or below	5589	4004 (71.6)	866 (15.5)	719 (12.9)	
High school	5222	3631 (69.5)	987 (18.9)	604 (11.6)	
University or above	7912	5022 (63.5)	1858 (23.5)	1032 (13.0)	
Sleep duration					<0.001
Very short	6560	4140 (63.1)	1480 (22.6)	940 (14.3)	
Short	5262	3636 (69.1)	1017 (19.3)	609 (11.6)	
Recommended	4529	3347 (73.9)	742 (16.4)	440 (9.7)	
Long	2372	1534 (64.7)	472 (19.9)	366 (15.4)	
Physical activity					<0.001
≤2h/day	16044	10898 (67.9)	2974 (18.6)	2172 (13.5)	
>2h/day	2679	1759 (65.7)	737 (27.5)	183 (6.8)	
Internet surfing					0.612
≤2h/day	3399	2321 (68.3)	664 (19.5)	414 (12.2)	
>2h/day	15324	10336 (67.4)	3047 (19.9)	1941 (12.7)	
Watching TV					0.079
≤2h/day	5065	3488 (68.9)	963 (19.0)	614 (12.1)	
>2h/day	13658	9169 (67.1)	2748 (20.1)	1741 (12.8)	
Playing on phone					<0.001
≤2h/day	1796	1114 (62.0)	213 (11.9)	469 (26.1)	
>2h/day	16927	11543 (68.2)	3498 (20.7)	1886 (11.1)	
Eating habits before sleeping					<0.001
Yes	14827	10249 (69.1)	2698 (18.2)	1880 (12.7)	
No	3896	2408 (61.8)	1013 (26.0)	475 (12.2)	

### Proportion of potential overweight/obesity influencing factors among children and adolescents according to different groups

Tables 2 and 3 showed the proportions and trend analysis results of potential overweight/obesity influencing factors among children and adolescents at different gender and different educational stages respectively. We found sleep duration, physical activity, internet surfing, watching TV, playing on phone, eating habits before sleeping were all tested with statistically significant difference among the three groups according to different gender and different educational stages respectively (all *P*<0.05). In addition, in Table 2, trend analyses of sleep duration with gender found the association between sleep duration and overweight/obesity at different gender showed a tendency, with the decreased of the sleep duration, the proportions of overweight/obesity in boys and girls were gradually increased (all *P*_trend_<0.05). However, in Table 3, we found no tendency between sleep duration and overweight/obesity at different educational stages.

**Table 2:** Proportion of potential overweight/obesity influencing factors among children and adolescents at different gender

Variables	Boys				Girls			
	Normal weight	Overweight	Obesity	*P*-value	Normal weight	Overweight	Obesity	*P*-value
Sleep duration				0.007				0.002
Very short	2400 (35.1)	764 (40.2)	645 (44.6)		1740 (29.9)	716 (39.6)	295 (32.5)	
Short	1980 (28.9)	539 (28.3)	386 (26.7)		1656 (28.5)	478 (26.4)	223 (24.6)	
Recommended	1763 (25.8)	335 (17.6)	238 (16.4)		1584 (27.2)	407 (22.5)	202 (22.2)	
Long	699 (10.2)	264 (13.9)	178 (12.3)		835 (14.4)	208 (11.5)	188 (20.7)	
*P*_trend_	0.035	0.028	0.017		0.042	0.031	0.025	
Physical activity				0.003				<0.001
≤2h/day	6200 (90.6)	1480 (77.8)	1300 (89.8)		4698 (80.8)	1494 (82.6)	872 (96.0)	
>2h/day	642 (9.4)	422 (22.2)	147 (10.2)		1117 (19.2)	315 (17.4)	36 (4.0)	
Internet surfing				0.012				0.017
≤2h/day	1319 (19.3)	339 (17.8)	252 (17.4)		1002 (17.2)	325 (18.0)	162 (17.8)	
>2h/day	5523 (80.7)	1563 (82.2)	1195 (82.6)		4813 (82.8)	1484 (82.0)	746 (82.2)	
Watching TV				0.014				0.023
≤2h/day	1994 (29.1)	444 (23.3)	388 (26.8)		1494 (25.7)	519 (28.7)	226 (24.9)	
>2h/day	4848 (70.9)	1458 (76.7)	1059 (73.2)		4321 (74.3)	1290 (71.3)	682 (75.1)	
Playing on phone				0.005				<0.001
≤2h/day	840 (12.3)	124 (6.5)	374 (25.8)		274 (4.7)	89 (4.9)	95 (10.5)	
>2h/day	6002 (87.7)	1778 (93.5)	1073 (74.2)		5541 (95.3)	1720 (95.1)	813 (89.5)	
Eating habits before sleeping				0.011				0.018
Yes	5599 (81.8)	1339 (70.4)	1066 (73.7)		4650 (80.0)	1359 (75.1)	814 (89.6)	
No	1243 (18.2)	563 (29.6)	381 (26.3)		1165 (20.0)	450 (24.9)	94 (10.4)

**Table 3:** Proportion of potential overweight/obesity influencing factors among children and adolescents at different educational stages

Variables	Primary school				Middle school				High school			
	Normal weight	Overweight	Obesity	*P*-value	Normal weight	Overweight	Obesity	*P*-value	Normal weight	Overweight	Obesity	*P*-value
Sleep duration				0.015				0.029				0.018
Very short	1184 (34.0)	592 (23.8)	347 (30.4)		1579 (34.4)	500 (80.8)	212 (30.6)		1367 (29.9)	374 (62.4)	405 (67.6)	
Short	479 (13.7)	978 (39.2)	255 (22.3)		2470 (53.8)	51 (8.3)	271 (44.1)		673 (14.7)	2 (0.3)	83 (13.9)	
Recommended	1043 (29.9)	572 (23.0)	277 (24.3)		527 (11.5)	30 (4.8)	14 (2.3)		1787 (39.0)	170 (28.3)	109 (18.2)	
Long	782 (22.4)	350 (14.0)	263 (23.0)		14 (0.3)	38 (6.1)	117 (19.0)		752 (16.4)	54 (9.0)	2 (0.3)	
*P*_trend_	0.424	0.586	0.387		0.521	0.497	0.286		0.527	0.561	0.435	
Physical activity				0.035				0.029				0.022
≤2h/day	2738 (78.5)	1955 (78.5)	1030 (90.2)		3821 (83.2)	499 (80.6)	587 (95.6)		4339 (94.8)	520 (86.7)	555 (92.7)	
>2h/day	750 (21.5)	537 (21.5)	112 (9.8)		769 (16.8)	120 (19.4)	27 (4.4)		240 (5.2)	80 (13.3)	44 (7.3)	
Internet surfing				0.021				0.027				0.033
≤2h/day	614 (17.6)	411 (16.5)	195 (17.1)		798 (17.4)	84 (13.6)	122 (19.9)		909 (19.9)	169 (28.2)	97 (16.2)	
>2h/day	2874 (82.4)	2081 (83.5)	947 (82.9)		3792 (82.6)	535 (86.4)	492 (80.1)		3670 (80.1)	431 (71.8)	502 (83.8)	
Watching TV				0.015				0.009				0.006
≤2h/day	755 (21.6)	549 (20.0)	328 (28.7)		1351 (29.4)	269 (43.5)	178 (29.0)		1382 (30.2)	145 (24.2)	108 (18.0)	
>2h/day	2733 (78.4)	1943 (78.0)	814 (71.3)		3239 (70.6)	350 (56.5)	436 (71.0)		3197 (69.8)	455 (75.8)	491 (82.0)	
Playing on phone				0.002				0.011				<0.001
≤2h/day	313 (9.0)	99 (4.0)	105 (9.2)		267 (5.8)	108 (17.4)	105 (17.1)		534 (11.7)	6 (1.0)	259 (43.2)	
>2h/day	3175 (91.0)	2393 (96.0)	1037 (90.8)		4323 (94.2)	511 (82.6)	509 (82.9)		4045 (88.3)	594 (99.0)	340 (56.8)	
Eating habits before sleeping				0.024				0.019				0.008
Yes	2634 (75.5)	1636 (65.7)	960 (84.1)		3390 (73.9)	517 (83.5)	443 (72.1)		4225 (92.3)	545 (90.8)	477 (79.6)	
No	854 (24.5)	856 (34.3)	182 (15.9)		1200 (26.1)	102 (16.5)	171 (27.9)		354 (7.7)	55 (9.2)	122 (20.4)	

### Association between sleep duration and risk of overweight/obesity

To be specific, multivariate logistic regression models were employed to analysis the direct associations between sleep duration and risk of overweight/obesity status at different gender and different educational stages in Tables 4 and 5. After adjustment for covariates, we found children and adolescents with very short sleep duration and short sleep duration may increase the risk of overweight/obesity, the association differed based on the gender-specific and educational stages-specific.

**Table 4:** Logistic regression analysis between sleep duration and overweight/obesity by gender

Variables	Crude OR(95%CI)	Adjusted OR^a^(95%CI)
Boys		
Sleep duration		
Very short	2.06 (1.23-2.98)	2.56 (1.34-3.16)
Short	1.78 (1.08-2.75)	2.13 (1.25-2.79)
Recommended	1.00(reference)	1.00(reference)
Long	0.58 (0.32-1.16)	0.75 (0.41-1.82)
Girls		
Sleep duration		
Very short	1.95 (1.15-1.97)	2.34 (1.26-2.88)
Short	1.72 (1.21-1.89)	2.09 (1.19-2.75)
Recommended	1.00(reference)	1.00(reference)
Long	0.45 (0.23-1.19)	0.68 (0.42-1.53)

^a^Adjusted OR were adjusted for age, residence region, height, weight, physical activity time, sedentary behaviors time, eating habits, parental age, parental BMI and parental educational level

**Table 5:** Logistic regression analysis between sleep duration and overweight/obesity by educational stages

Variables	Crude OR(95%CI)	Adjusted OR^a^(95%CI)
Primary school		
Sleep duration		
Very short	1.82 (1.23-2.14)	2.15 (1.17-2.66)
Short	1.27 (1.16-1.35)	1.69 (1.34-1.92)
Recommended	1.00(reference)	1.00(reference)
Long	0.42 (0.31-1.26)	0.53 (0.31-1.17)
Middle school		
Sleep duration		
Very short	1.67 (1.34-1.88)	2.26 (1.21-2.83)
Short	1.18 (1.09-1.35)	1.58 (1.17-1.79)
Recommended	1.00(reference)	1.00(reference)
Long	0.51 (0.28-1.24)	0.65 (0.31-1.24)
High school		
Sleep duration		
Very short	1.91 (1.41-2.27)	2.23 (1.27-2.86)
Short	1.33 (1.22-1.55)	1.51 (1.17-1.89)
Recommended	1.00(reference)	1.00(reference)
Long	0.56 (0.32-1.31)	0.69 (0.35-1.57)

^a^Adjusted OR were adjusted for age, residence region, height, weight, physical activity time, sedentary behaviors time, eating habits, parental age, parental BMI and parental educational level

## Discussion

Overweight and obesity has become an international epidemic as the risk of overweight/obesity-related morbidity increases in different countries among children and adolescents. Considering the long-term potential complications of overweight/obesity, identifying the different related risk factors is increasingly underscored. Using a cross-sectional survery on 18723 children and adolescents aged 6-17 years in Henan Province, we found that the proportions of overweight and obesity were 19.8% and 12.6% respectively. The levels found here were higher than the proportion previously reported among students aged 7-18 years in China from 1985 to 2014, where 19.4% of survey participants were overweight/obesity [23]. It indicates that the overweight and obesity problem of children and adolescents in Henan Province is facing the same severe situation as that of the whole country, and it is necessary to take targeted measures to intervene and control.

Generally, the results of this study showed that the proportions between sleep duration, related lifestyle behaviors and overweight/obesity were gender- and educational stages-specific. In the total population, the proportions of overweight/obesity in boys were significantly higher than those of girls. When evaluated by gender, the proportions of overweight/obesity were higher in boys than girls under different sleep duration. In addition, trend analyses found with the decreased of the sleep duration, the proportions of overweight/obesity in boys and girls were gradually increased. Except for differences between samples in the studies conducted, it is thought that the higher proportion of overweight/obesity in boys may be related with genetics, environment, family and school behaviors, boys may spend more time in screen-based sedentary behaviors associated with less physical activity. Lower proportion of overweight/obesity in girls can also be associated with the fact that, overall, girls need and consume fewer calories than boys and they are more conscious of their body shape control in daily life. This reminds that gender eapecially boys can present an interesting population for further investigation. In terms of gender differences, to control the proportion of overweight/obesity, boys in particular may be susceptible to unhealthy behaviors and preventative measures tailored for boys may help to address this issue. Additionally, we found the proportion of overweight/obesity in primary school were higher than those of middle school and high school. It may be relate with primary school students have a low level of overweight/obesity health education knowledge, and are vulnerable to the temptation of delicious food under a poor self-control. However, middle and high school students are relatively more self-disciplined and can better control their eating habits and sleep related lifestyle behaviors. Therefore, parents and schools should combine the characteristics of gender- and educational stages-specific in children and adolescents, and improve the situation of healthy behaviors, so as to reduce the occurrence of overweight/obesity.

In this study, after adjustment for confounders, we observed students with very short sleep duration and short sleep duration were significantly associated with an increased risk of overweight/obesity according to different gender and different educational stages. Our findings are consistent with the previous epidemiological studies [24–28] that reported relationships between the risk of overweight/obesity and sleep duration and related lifestyle behaviors. Several possible explanations [29, 30] have been put forward to explain the mechanism of the associations between sleep duration and overweight/obesity. The hormones is currently considered the most plausible reason for the increased risk of overweight/obesity of short duration sleepers [31]. Laboratory studies revealed that short sleep could increase ghrelin level and decrease leptin level in the body, which may alter eating habits and eventually predispose overweight/obesity in the future [32]. Additionally, sedentary behaviors, such as internet surfing, watching TV or playing on phone, proved to be important lifestyle factors of children and adolescents overweight/obesity. Reduction in sleep duration and increase in sedentary time or physical activity among children and adolescents may intercorrelate. Very short sleep may also cause children and adolescents to sleep more during the day, thus reducing their daytime physical activity and causing weight gain. Since short sleep duration and related lifestyle behaviors are modifiable risk factors, these findings are instructive in the clinical prevention and treatment of children and adolescents overweight/obesity.

Several possible limitations should be considered in the present study. Firstly, given that the study was conducted in a cross-sectional design, the causal pathways underlying the observed relationships could hardly be verified. Further cohort studies are needed if permitted. Secondly, the sleep duration and related lifestyle behaviors data in the study were obtained by a self-reported method. While these data generally correspond well with more objective measures, the potential for inaccuracies and biases may be inescapable. Thirdly, daytime napping and sleepiness, the differences between weekdays and weekends in sleep duration were not covered by our questionnaire. Additionally, we could not analyze the data for sleep quality which is considered to play an important role in developing overweight/obesity. Finally, though our present study included a large sample with a wide range of ages in Henan province, the results might not be representative of the national population in China.

## Conclusions

In conclusion, children and adolescents aged 6-17 years had a high proportion of overweight/obesity in Henan Province. Very short sleep duration and short sleep duration may increase the risk of overweight/obesity, the association differed based on the gender-specific and educational stages-specific. Data suggest that gender and educational stages should be regarded as specific characteristics for the effects on overweight/obesity. Further research should clarify the gender- and educational stages-specific causal effects and explore the underlying mechanisms. Our findings may have some implications for practical intervention programs and policies to prevent children and adolescents unhealthy related behaviors and the occurrence of overweight/obesity.

## Data Availability

The datasets used and/or analysed during the current study are available from the corresponding author on reasonable request.

## Abbreviations

ANOVA: One-way analysis of variance
ORs: Odds ratios
CIs: Confidence intervals
